# Mammalian Target of Rapamycin (mTOR) Signaling at the Crossroad of Muscle Fiber Fate in Sarcopenia

**DOI:** 10.3390/ijms232213823

**Published:** 2022-11-10

**Authors:** Giuseppe Sirago, Anna Picca, Riccardo Calvani, Hélio José Coelho-Júnior, Emanuele Marzetti

**Affiliations:** 1 Department of Biomedical Sciences, Università degli Studi di Padova, 35131 Padua, Italy; 2 Fondazione Policlinico Universitario “Agostino Gemelli” IRCCS, 00168 Rome, Italy; 3 Department of Medicine and Surgery, LUM University, 70100 Casamassima, Italy; 4 Department of Geriatrics and Orthopedics, Università Cattolica del Sacro Cuore, 00168 Roma, Italy

**Keywords:** aging, skeletal muscle, autophagy, mitophagy, protein synthesis, protein degradation, atrogenes, neuromuscular junction, rapalogs, calorie restriction

## Abstract

The mammalian target of rapamycin (mTOR) is a major regulator of skeletal myocyte viability. The signaling pathways triggered by mTOR vary according to the type of endogenous and exogenous factors (e.g., redox balance, nutrient availability, physical activity) as well as organismal age. Here, we provide an overview of mTOR signaling in skeletal muscle, with a special focus on the role played by mTOR in the development of sarcopenia. Intervention strategies targeting mTOR in sarcopenia (e.g., supplementation of plant extracts, hormones, inorganic ions, calorie restriction, and exercise) have also been discussed.

## 1. Introduction

Sarcopenia, the age-related decline of muscle mass and strength/function is a major risk factor for disability and loss of independence in late life [1]. Studies have shown that behavioral interventions (e.g., physical activity, adapted nutrition) reduce the rate of muscle wasting during aging [2]. However, an incomplete understanding of the mechanisms driving age-related muscle loss has hampered the development of effective drugs to prevent or treat sarcopenia. Altered muscle protein metabolism is considered to be one of the main factors underlying the development and progression of sarcopenia [3]. While basal rates of muscle protein synthesis (MPS) and degradation (MPD) seem to be unaffected by age [4], the anabolic response to a variety of stimuli (e.g., exercise, nutrient ingestion) is blunted during aging [4]. The mammalian target of rapamycin (mTOR) is a key regulator of muscle anabolic and catabolic pathways and, hence, a promising target for interventions against sarcopenia [5].

Rapamycin, a molecule with antifungal, immunosuppressive, and antiproliferative properties, has come into the spotlight during the last decades as a candidate drug for targeting sarcopenia [6]. However, the clinical use of rapamycin and synthetic rapalogs to manage sarcopenia is far from being achieved, mostly because of the lack of organ-targeted drug delivery and the risk of systemic side effects. Here, we provide an overview of the role of mTOR signaling in muscle protein metabolism and its implications in sarcopenia, as well as a description of current strategies being tested that harness this signaling pathway to manage sarcopenia.

## 2. Supramolecular Organization of the Mammalian Target of Rapamycin

Growing evidence points to the mTOR pathway as a major regulator of skeletal myofiber viability. This serine-threonine kinase is at the crossroad of cell proliferation and survival and, through the supramolecular mTOR complexes mTORC1 (mammalian target of rapamycin complex 1) and mTORC2, controls muscle fiber metabolism, growth, and proliferation [7]. Cryo-electron microscopy resolution experiments have revealed the supramolecular organization of the two mTOR complexes. In particular, mTORC1 appears to be organized into three main core proteins (i.e., mTOR, Raptor, and mammalian homolog of protein lethal with sec 13 protein 8), and two accessory regulatory proteins (i.e., GβL and Deptor) [7,8]. mTORC2, instead, seems to be organized into four main core proteins (i.e., mTOR, Rictor, the stress-activated map kinase-interacting protein 1 Sin1 (mSIN1), and mLST8), with three accessory regulatory proteins (i.e., PRR5/Protor-1, DEPTOR, and GβL) [7,9,10]. The molecular characterization of these supramolecular machineries is still incomplete and novel functions have recently been described. For instance, a direct role of Raptor in stimulating mitochondrial protein synthesis has been reported during muscle hypertrophy in mice [11]. It is noteworthy that the mTOR structure adapts to the myocyte metabolic milieu via chemical modifications and/or interactions with other protein complexes to modulate cell metabolism [7]. In the next sections, the integration of anabolic and catabolic signals by mTOR in the setting of muscle metabolism is described.

## 3. Muscle Protein Synthesis: The Mammalian Target of Rapamycin Complex 1 Axis

Muscle growth is regulated by several stimuli, such as mechanical loading and calcium uptake during physical exercise, insulin, insulin growth factor 1 (IGF-1), and amino acid signaling, that ultimately activate the phosphatidyl inositol 3-kinase (PI3K)–serine/threonine kinase 1 (AKT)–mTORC1 axis [3,12]. This axis stimulates protein synthesis and inhibits proteolysis, thereby switching muscle protein metabolism towards MPS and preserving cellular homeostasis in a balance with autophagy. Such modulation has been observed along with the stabilization of the neuromuscular junction (NMJ) and allows efficient excitation–contraction coupling [13,14,15]. Reduced mTORC1 signaling in murine models has been reported to trigger fiber denervation with the appearance of myofibers positive for neural cell adhesion molecules and segmented NMJ morphology [15]. This indicates that skeletal muscle fibers may be able to exert direct control over innervation to model motor neuron plasticity in response to molecular and metabolic changes. Non-coding RNAs (ncRNAs) are emerging as important regulators of muscle metabolism, and miR-29c seems to be able to trigger MPS and block MPD independent of the mTOR axis [16]. Conversely, mir-145-5p may inhibit mTOR phosphorylation, resulting in its deactivation [17].

### mTORC1 Axis: Different Stimuli for Muscle Protein Synthesis

The two main upstream activators of mTOR (i.e., amino acids/glucose and insulin/IGFs) operate via specific signaling pathways depending on the type of stimulus [18,19]. Upon binding with their sarcolemmal receptors, insulin and IGF-1 trigger differential signaling pathways. In particular, the insulin receptor preferentially activates mTORC1 and AKT signaling to regulate metabolism, while the IGF-1 receptor preferentially activates Rho GTPases to regulate cellular growth [20]. mTORC2, instead, controls cell proliferation and survival via phosphorylation and activation of AKT [21].

Physical exercise stimulates PI3K signaling via integrins, located in the sarcolemma, which receive and integrate mechanical stimuli [22]. These are then converted into phosphorylation of focal adhesion kinases, mainly anchored to integrins at the sarcoplasmic side of the sarcolemma, ultimately resulting in the activation of the PI3K–phosphoinositide-dependent kinase 1–AKT–mTOR cascade [22]. Raptor seems to be the main factor phosphorylated by PI3K signaling during exercise [23,24]. The differential activation of the mTOR pathway is accomplished via a molecular feedback loop that blocks the activation of AKT upon mTORC1 and, therefore, represents a checkpoint for muscle growth or proteostasis [7]. The modulation of Raptor by exercise indicates that mTORC1 may control MPS under an intense rate of contraction, but not at a basal rate, or at least it could not be the only factor involved, which would explain its apparent loss of function during aging [24].

Nutrients, such as glucose and amino acids, are also able to activate protein translation and promote MPS via mTOR [25,26]. In particular, amino acids can activate mTORC1 through four small guanosine triphosphatases (GTPases), called Rag GTPases A–D, that bind Raptor and also activate Rheb, another GTP-binding protein, resulting in the activation of mTOR through a Rheb-GTP-binding-dependent mechanism [27]. The exact mechanism through which Rag activates mTOR is not well understood. However, Rag interaction with Raptor seems to be strongly inhibited in catabolic conditions, such as alcohol abuse and sarcopenia [28].

## 4. Muscle Protein Degradation: The Other Face of the mTORC1 Axis during Aging

**Figure 1:**
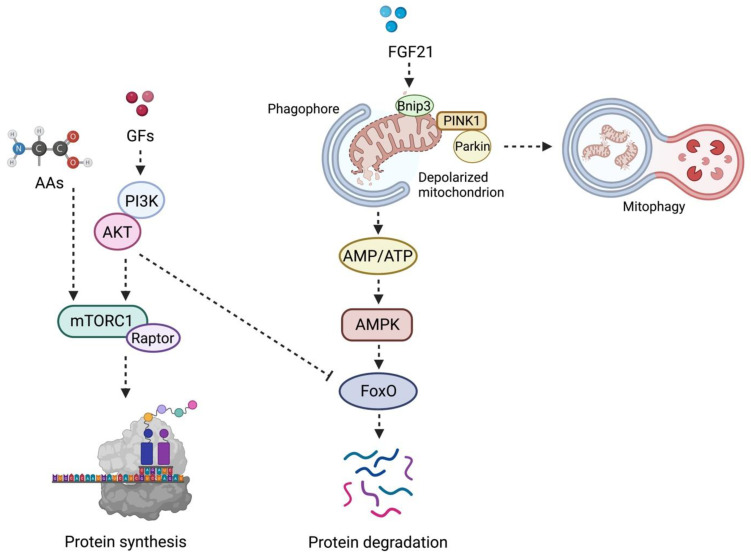
Schematic Representation of the Coordinated Regulation of the Mammalian Target of Rapamycin and Mitophagy in Muscle Protein Synthesis and Degradation. In the presence of growth factors (e.g., insulin, insulin-like growth factor 1, growth hormone), the phosphoinositide 3-kinases-protein kinase B (PI3K) is activated and triggers muscle protein synthesis via the mammalian target of rapamycin complex 1 (mTORC1). The latter complex is also positively modulated by amino acid availability. Conversely, downregulation of PI3K signaling induces translocation of Forkhead box O (FoxO) into the nucleus, where it regulates the transcription of the ubiquitin-ligases muscle ring finger 1 (MuRF1) and muscle atrophy F-box (MAFbx) genes. The activation of this signaling pathway induces degradation of sarcomere components and ignites a muscle pro-atrophy response. The same degradative molecular program is also triggered by fibroblast growth factor 21 (FGF21) in the setting of mitochondrial dysfunction and oxidative stress. In this case, the release of FGF21 stimulates the expression of the mitophagy-related protein B-cell lymphoma 2 interacting protein 3, paralleled by recruitment of the phosphatase and tensin homolog-induced kinase 1 (PINK1) through the translocases of the inner and the outer membranes and its activation at the site of depolarized mitochondria. This event promotes the sequestration of the E3 ubiquitin ligase Parkin at the outer mitochondrial membrane and guides the clearance of dysfunctional organelles. Finally, depolarized mitochondria are coated and prepared for disposal by the ubiquitin-binding adaptor protein p62/sequestosome-1 and the recruitment of the microtubule-associated proteins 1A/1B light chain 3B (LC3). This enables the transfer of mitochondria to lysosomes. FoxO-dependent atrophy is also pursued when severely damaged and bioenergetically incompetent mitochondria are not efficiently removed and, thus, the AMP/ATP ratio increases, which engages 5′ AMP-activated protein kinase (AMPK). Abbreviations: AA, amino acid; AMP, adenosine monophosphate; ATP, adenosine triphosphate; GF, growth factor; TIM23, translocase of the inner membrane 23; TOM, translocase of the outer membrane. Created with BioRender.com, accessed on 26 August 2022.

The PI3K–AKT–mTORC1 axis can trigger MPD via AKT signaling, especially during aging and in the setting of oxidative stress [29] (Figure 1). AKT is a serine-threonine protein kinase that regulates cell proteostasis, apoptosis, and turnover [29]. Upon activation, AKT phosphorylates Forkhead box protein O (FoxO) transcriptional regulators, thereby limiting their access to the nucleus and resulting in downregulation of atrogenes expression.

Atrogenes are E3 ubiquitin ligases that catalyze the rate-limiting step of the ubiquitin–proteosome system and allow recognition of substrates to be degraded. Muscle RING-finger 1 (MuRF1) and muscle atrophy F-box (Atrogin-1/MAFbx) are the two muscle-specific ubiquitin ligases. Among other emerging E3 ubiquitin ligases, Trim32 is specifically involved in the degradation and renewal of skeletal muscle sarcomere components [30]. Trim32 mutations have been found in limb–girdle muscular dystrophy 2H, and mice knock-out for Trim32 develop premature sarcopenia [31]. The maintenance of adequate levels of Trim32 has been associated with the preservation of muscle mass, reinnervation capacity, and NMJ plasticity during human aging [32]. A balance between AKT activation and repression is instrumental in achieving muscle protein homeostasis and preventing the accrual of defective unfolded proteins. Polymorphisms in FoxO genes have been linked to longevity and are included among the genetic mechanisms contributing to aging [33]. The existence of genetic variants and/or pools of genetic variants can also explain, at least partly, the variability existing among older adults in muscle responses to internal or external stimuli and, ultimately, in the susceptibility to develop sarcopenia [34].

Finally, sestrins, a class of biomolecules produced in response to stressful conditions, have also been described as relevant regulators of anabolic and degradative pathways orchestrated by mTORC1 [35]. In flies, sestrins are crucial for detecting leucine-containing food [36]. Sestrin-null flies lose the ability to detect leucine-free food and are unable to preferentially feed the progeny with leucine-containing food [36]. This ability is conveyed to flies by the capacity of sestrins to bind leucine. The latter is the main regulator of mTORC1 activity, and this nutrient-sensing role of mTORC1 is pivotal for mediating not only detection, but also adaptation to low-leucine diets in Drosophila [36]. Sestrin1, the skeletal muscle isoform of sestrin, shows a high affinity for leucine. Sestrin1 can bind to a complex named GTPase-activating protein activity toward Rags 2 (GATOR2), which sequesters Sestrin1. Leucine-induced activation of mTORC1 is directly controlled by the dissociation of Sestrin1 from GATOR2, due to the high affinity of Sestrin1 for leucine [37].

Together with ncRNAs, these stress-induced molecules represent emerging factors that may be able to modulate the mTOR pathway and that could, therefore, be exploited for drug development.

## 5. The mTORC2 Axis: Not a Twin Complex

Similar to the role exerted by Raptor in the mTORC1 complex, mTORC2 relies on the core subunits Rictor and mSIN1 (also known as mitogen-activated protein kinase 2-associated protein 1 (MAPKAP1)) for its regulation [9,10,38]. However, the two mTOR complexes have a different influence on skeletal myocyte fate. Indeed, muscle-specific ablation of Raptor (i.e., mTORC1 deactivation) induces progressive muscle dystrophy and atrophy [38]. Conversely, ablation of Rictor (i.e., mTORC2 deactivation) does not cause muscle atrophy, such that the lack of both genes leads to a muscle phenotype that does not differ from that of Raptor knockout [38,39]. The set of ultrastructural changes observed in muscle fibers following mTORC1 ablation includes infiltration of immune cells, centralized myonuclei, and altered microscopic muscle architecture with aberrant muscle fiber distribution [38]. The two complexes do not appear to influence one another, as shown by the downregulation of Rictor in C2C12 myoblasts [40]. However, the last stages of myogenic differentiation seem to also involve mTORC2, at least in vitro [38,40].

Albeit mTORC2 may not be crucial for skeletal myocyte viability, experiments in murine models have indicated that this complex is among the molecular factors involved in enhancing muscle performance [41,42]. Such a role is deployed during the over-expression of retinol dehydrogenase (SRP35/DHRS7C), an enzyme involved in retinol metabolism, or during Sestrin2 overexpression [41,42]. Indeed, muscle stem cells show dependence on mTORC2 signaling under stressful conditions, such as cardiotoxin administration and aging [43]. This suggests that mTORC2 might play relevant roles in late life. mTORC2 is also involved in mediating the myotoxic effects of simvastatin administration [44].

## 6. mTOR Axis in Sarcopenia: All That Glitters Is Not Gold

Sarcopenia is characterized by anabolic resistance even in the setting of chronic mTOR activation [14,29,45,46,47]. A shift of muscle fiber composition towards slow-twitch fibers and reduced expression of the glucose transporter GLUT4 in fast-twitch fibers have been described in older adults, accompanied by the reduced ability of the muscle to utilize glucose upon insulin stimulation [48,49]. This is often associated with metabolic disorders for which the modulation of insulin signaling is altered (e.g., obesity, diabetes). Sarcopenia can exacerbate these conditions and be a contributor and/or a consequence, being the skeletal muscle an endocrine organ releasing active molecules that control whole-body metabolism. Such a role has been described also in patients with liver cirrhosis, in whom alterations in the mTOR pathway and autophagy are indicated as mediators of sarcopenia [50].

Studies in preclinical models have shown that the ability to metabolize glucose in muscle seems to be more affected by age-associated dietary changes than muscle metabolism itself, even though insulin resistance can exacerbate muscle wasting [51]. Sarcopenia is also frequently associated with reduced circulating levels of IGF-1, which has been indicated as a possible biomarker for the condition [52,53,54,55]. Conversely, the expression of the IGF-1 receptor increases with aging, possibly as a compensatory response to lower IGF-1 levels, leaving open the question as to why mTOR activation is enhanced in sarcopenia [47]. Several factors, including physical activity and genetic background, can modulate mTOR signaling at the transcriptional level via microRNAs. In the context of sarcopenia, this pathway may also be modulated depending on muscle type and age/sex, as it occurs in neuromuscular adaptations to unloading [56]. Thus, specific molecular signatures of muscle aging other than the molecular pathways elicited by physical activity exist and warrant investigation [57,58,59,60].

The formation of mTORC1/2 complexes is pivotal for the activation of the mTOR pathway. Altered interactions between Raptor, Deptor, and mTOR have been implicated in MPS reduction in mice challenged with catabolic stimuli [28]. MPS requires the assembly and coordination of an intricated translation machinery within the sarcoplasm or at the sarcoplasmic reticulum [61]. More specifically, the eukaryotic translation initiation factor 4E (eIF4E) associates with the eIF4G1/eIF4G3 complex and triggers protein translation [61]. MPS is eventually accomplished following sequestration and phosphorylation of eIF4E-binding protein 1 (eIF4E-BP1) and of ribosomal S6 protein by the ribosomal S6 kinase 1 (S6K1) upon mTORC1 activation [61]. During aging, the mTOR axis becomes hyperactive with a consequent increase in the levels of phosphorylated eIF4E-BP1, S6 protein, S6K1, and mTORC1 [62,63,64]. However, this mTORC1 activation is unable to increase basal MPS in older adults or in old mice in the fasted state [60,65,66]. Muscle anabolic response has also been reported to be delayed during aging even under stimuli such as exercise or essential amino acid ingestion [60,65,66].

Therefore, although higher mTOR activation can be observed during sarcopenia following anabolic stimuli, this signal may not promote the correct assembly of the protein translation machinery, resulting in insufficient MPS [67]. Along the same line of evidence, results gathered from studies conducted in aged rats showed that an increase in mTOR-dependent protein synthesis was not paralleled by muscle mass accrual, possibly due to age-associated chronic catabolic insults [68]. Conversely, the detrimental effect of a chronic hyperphosphorylation state of mTOR on muscle could be rescued by treatment with rapamycin which was shown to set the muscle in a “healthy state” via S6K1 and eIF4E-BP1 signaling, at least in rapamycin-resistant double-knockout mice [69]. Interestingly, the eIF4E-BP1 and eIF4E-BP2 complexes have also been indicated as candidate targets for the management of sarcopenia [70]. The beneficial effects of rapamycin treatment and reduced mTOR response in sarcopenic muscles may involve decreased mTOR activation mediated by the IGF-binding protein 7 (Igfbp7)–AKT–mTOR axis [71]. Igfbp7 binds the IGF receptor to block its activation, which is required by muscle satellite cells to avoid exhaustion and probably favor muscle regeneration [71]. ncRNAs that modulate the mTOR pathway have been included among the molecules composing the transcriptional network that characterizes the skeletal muscle of physically inactive older adults [72]. In particular, the microRNAs miR-29c and mir-145-5p are mTOR modulators [16,17]. A protective role for sestrins toward the development of sarcopenia has also been reported [35]. Whether an increase in MPD occurs in sarcopenia is still debated. Indeed, either increases or decreases in atrogenes expression in murine models of aging have been described [47,73,74,75]. Some authors attribute such a discrepancy to differences in the muscles analyzed and, thus, fiber type composition. However, no changes in mRNA or protein content of atrogenes have been reported in humans [46,76,77,78,79,80]. Thus, the role of these pathways in sarcopenia warrants further investigation. 

Taken as a whole, current evidence indicates that, while the mTOR pathway is stimulated to achieve an increase in MPS and reduce MPD in sarcopenia, this does not translate into a preservation of muscle mass. These findings are in keeping with enhanced AKT signaling that is not paralleled by changes in the expression of atrogenes in humans. Nevertheless, sarcopenic muscles are still able to respond to specific treatments (e.g., rapamycin). Therefore, a deeper understanding of the molecular mechanisms conveying such a response may help identify pathways of muscle resilience and discover novel targets for drug development.

## 7. Current Strategies Targeting mTOR to Manage Sarcopenia

Multiple factors, including environmental stressors, diet, and physical activity, influence the decline of skeletal muscle health and may evoke muscle-wasting signals. For instance, chemical compounds released by microplastics, such as di-(2-ethyl hexyl) phthalate, have been found to trigger oxidative stress and myonuclear apoptosis via inhibition of the PI3K–AKT–mTOR pathway [81]. However, the mTOR pathway can also be positively regulated. Studies reported in Table 1 show that, despite the controversial role of mTOR in sarcopenia, mTOR modulation in response to lifestyle interventions and/or specific drugs may prevent or block age-related muscle wasting.

Physical exercise is the best-studied intervention and the most efficacious against muscle decay. Molecular insights on the mechanisms by which exercise elicits beneficial effects in sarcopenia have been reported by Zeng et al. [82] who showed that downregulation of mTOR signaling, together with stimulation of autophagy, can renovate muscle structures and ameliorate the overall quality of cell organelles.

As for dietary interventions, both calorie restriction and specific food sources, such as fish protein and inorganic ions (e.g., magnesium) or some types of fermented milk, have been reported to be beneficial for muscle health and to act via mTOR axis activation [83,84,85]. Approximately 27% of magnesium is stored within the skeletal muscle where it participates to MPS via mTOR, muscle relaxation, energy supply in form of ATP, glycogen breakdown, fat oxidation, oxygen uptake, and electrolyte balance [84]. Amino acid supplementation has also been widely investigated as a strategy to favor muscle anabolism. However, differential responses have been reported according to amino acid types. While the administration of non-essential amino acids is unable to evoke MPS, the infusion of essential amino acids has been proven to enhance muscle anabolism rates to up to 90%. Among essential amino acids, branched-chain amino acids and, in particular, leucine, are recognized as major triggers of muscle anabolism. mTOR and its downstream effectors are the main molecular target of branched-chain amino acids, including leucine [36].

Calorie restriction, the reduction of total calorie intake without malnutrition, is one of the best-known life-extending interventions [86]. Such an anti-aging effect is achieved also through the modulation of mTOR [86]. A hyper-phosphorylated state of mTOR has been identified in sarcopenia, and calorie restriction represses this age-associated hyper-signaling in rats [86]. Of note, the duration of treatment, as well as the age window of the intervention, may be relevant to establish the molecular mechanisms to be elicited and the beneficial effect evoked [87,88,89]. For instance, calorie restriction is unable to trigger hypo-regulation of mTOR in young animals, in which unaltered protein levels have been found [86]. Although additional studies are needed to gather more conclusive data, it seems that the sensitivity of skeletal muscle to treatments depends on the age window and treatment length, which need to be considered for devising interventional strategies that may be efficient in advanced age. The supplementation of resveratrol, a calorie restriction mimetic, in old age or a combination of calorie restriction plus resveratrol has been found to be beneficial for counteracting muscle wasting [90,91]. The beneficial effects of calorie restriction have also been demonstrated at the level of the brain, peripheral nerves, and liver [92,93,94]. However, the downside of reduced quality of life under a chronic or lifelong calorie restriction regimen has been documented, thus hampering its sustainability, and leaving to this therapeutic option the role of an experimental model to untangle the molecular mechanisms involved. 

Hormones, such as testosterone, have been shown to convey beneficial adaptations via the mTOR axis in old men engaged in resistance exercise programs [95]. Preclinical studies are also currently focusing on the use of synthetic peptides to control MPS, such as the LRS-UNE-L peptide and collagen hydrolysate tripeptides [96,97]. In particular, LRS-UNE-L has been shown to target the mTORC1-activating domain and enhance complex activity [97]. Finally, plant extracts and other natural compounds, including Chrysanthemum extracts, the root of Maca, and rimonabant, are under investigation for their potential to induce muscle hypertrophy by enhancing mTOR signaling [98,99,100].

**Table 1:** Strategies and ongoing preclinical studies targeting the mTOR pathway.

Strategy	Model	Reference	Main Findings
Lactococcus cremoris subsp. cremoris FC-fermented milk	Aged mouse	Aoi et al., 2022 [85]	Increase in mTOR phosphorylation and MPS
LRS-UNE-L peptide	C2C12 cells and aged mouse	Baek et al., 2022 [97]	Stimulation of mTORC1 axis and enhanced muscle fiber regeneration
Clinical trial on testosterone + resistance exercise	Men (65–75 years)	Gharahdaghi et al., 2019 [95]	Upregulation of mTOR signaling and increase in muscle mass and function
Collagen hydrolysate tripeptides (CTP)	Aged mouse	Kim et al., 2022 [96]	Stimulation of mTOR signaling and increase in muscle mass
Chrysanthemum extracts (CME)	Aged rat and mouse	Kwon et al., 2021 [100]	Increase in mTOR phosphorylation and amelioration of muscle mass and function
Rimonabant	C2C12 cells	Le Bacquer et al., 2021 [99]	Increase in mTOR phosphorylation and MPS
Magnesium	C2C12 cells and aged mouse	Liu et al., 2021 [84]	Increase in mTOR phosphorylation, enhanced muscle regeneration, preservation of muscle mass and strength
Fish proteins	Young rat	Morisasa et al., 2022 [83]	Promotion of muscle hypertrophy via AKT–mTOR signaling
Calorie restriction	Aged rat	Chen et al., 2019 [86]	Decrease in mTOR content/phosphorylation and preservation of muscle mass
Root of Maca	C2C12 cells	Yi et al., 2022 [98]	Promotion of muscle hypertrophy via AKT–mTOR signaling
Resistance exercise	Aged rat	Zeng et al., 2020 [82]	Decrease of mTOR signaling, stimulation of autophagy, preservation of muscle mass and function

## 8. Conclusions and Perspectives

The involvement of mTOR in sarcopenia is controversial. In particular, the implications of mTOR hyperphosphorylation during aging are not fully understood. Preclinical studies and small clinical trials have shown that specific compounds promote MPS via stimulation of mTOR. However, other studies reported that calorie restriction and resistance exercise may exert protective effects against sarcopenia through the downregulation of mTOR and stimulation of autophagy. These mixed findings may be explained, at least in part, by emerging studies indicating that, besides mTOR, additional routes that modulate MPS and MPD are under the control of ncRNAs. Furthermore, mTORC1 is an emerging player in the modulation of NMJ plasticity [15]. Thus, additional studies are needed to dissect the molecular mechanisms that control muscle–nerve communication and clarify the counterintuitive hyperactivation of mTOR in sarcopenia to identify pathways of muscle resilience and discover novel targets for drug development.

## Data Availability

Not applicable.

## References

[1] Marzetti E., Calvani R., Tosato M., Cesari M., Di Bari M., Cherubini A., Collamati A., D’Angelo E., Pahor M., Bernabei R. (2017). Sarcopenia: An Overview. Aging Clin. Exp. Res..

[2] Bernabei R., Landi F., Calvani R., Cesari M., Del Signore S., Anker S.D., Bejuit R., Bordes P., Cherubini A., Cruz-Jentoft A.J. (2022). Multicomponent Intervention to Prevent Mobility Disability in Frail Older Adults: Randomised Controlled Trial (SPRINTT Project). BMJ.

[3] Sartori R., Romanello V., Sandri M. (2021). Mechanisms of Muscle Atrophy and Hypertrophy: Implications in Health and Disease. Nat. Commun..

[4] Fry C.S., Rasmussen B.B. (2011). Skeletal Muscle Protein Balance and Metabolism in the Elderly. Curr. Aging Sci..

[5] Yoon M.S. (2017). mTOR as a Key Regulator in Maintaining Skeletal Muscle Mass. Front. Physiol..

[6] Tang H., Shrager J.B., Goldman D. (2019). Rapamycin Protects Aging Muscle. Aging.

[7] Saxton R.A., Sabatini D.M. (2017). MTOR Signaling in Growth, Metabolism, and Disease. Cell.

[8] Aylett C.H.S., Sauer E., Imseng S., Boehringer D., Hall M.N., Ban N., Maier T. (2016). Architecture of Human mTOR Complex 1. Science.

[9] Yu Z., Chen J., Takagi E., Wang F., Saha B., Liu X., Joubert L.M., Gleason C.E., Jin M., Li C. (2022). Interactions between mTORC2 Core Subunits Rictor and MSin1 Dictate Selective and Context-Dependent Phosphorylation of Substrate Kinases SGK1 and Akt. J. Biol. Chem..

[10] Scaiola A., Mangia F., Imseng S., Boehringer D., Berneiser K., Shimobayashi M., Stuttfeld E., Hall M.N., Ban N., Maier T. (2020). The 3.2-Å Resolution Structure of Human mTORC2. Sci. Adv..

[11] Baraldo M., Nogara L., Dumitras G.A., Dondjang A.H.T., Geremia A., Scalabrin M., Türk C., Telkamp F., Zentilin L., Giacca M. (2021). Raptor Is Critical for Increasing the Mitochondrial Proteome and Skeletal Muscle Force during Hypertrophy. FASEB J..

[12] Baehr L.M., Hughes D.C., Waddell D.S., Bodine S.C. (2022). SnapShot: Skeletal Muscle Atrophy. Cell.

[13] Vainshtein A., Sandri M. (2020). Signaling Pathways That Control Muscle Mass. Int. J. Mol. Sci..

[14] Ham D.J., Börsch A., Lin S., Thürkauf M., Weihrauch M., Reinhard J.R., Delezie J., Battilana F., Wang X., Kaiser M.S. (2020). The Neuromuscular Junction Is a Focal Point of MTORC1 Signaling in Sarcopenia. Nat. Commun..

[15] Baraldo M., Geremia A., Pirazzini M., Nogara L., Solagna F., Türk C., Nolte H., Romanello V., Megighian A., Boncompagni S. (2020). Skeletal Muscle mTORC1 Regulates Neuromuscular Junction Stability. J. Cachexia Sarcopenia Muscle.

[16] Ketilly P., Alves N., Cruz A., Silva W.J., Labeit S., Moriscot A.S. (2022). MiR-29c Increases Protein Synthesis in Skeletal Muscle Independently of AKT/mTOR. Int. J. Mol. Sci..

[17] Jin J., Li F., Fan C., Wu Y., He C. (2022). Elevated Mir-145-5p Is Associated with Skeletal Muscle Dysfunction and Triggers Apoptotic Cell Death in C2C12 Myotubes. J. Muscle Res. Cell Motil..

[18] Picca A., Calvani R., Sirago G., Coelho-Junior H.J., Marzetti E. (2021). Molecular Routes to Sarcopenia and Biomarker Development: Per Aspera Ad Astra. Curr. Opin. Pharmacol..

[19] Liu G.Y., Sabatini D.M. (2020). mTOR at the Nexus of Nutrition, Growth, Ageing and Disease. Nat. Rev. Mol. Cell Biol..

[20] Nagao H., Cai W., Albrechtsen N.J.W., Steger M., Batista T.M., Pan H., Dreyfuss J.M., Mann M., Kahn C.R. (2021). Distinct Signaling by Insulin and IGF-1 Receptors and Their Extra- And Intracellular Domains. Proc. Natl. Acad. Sci. USA.

[21] Sarbassov D.D., Guertin D.A., Ali S.M., Sabatini D.M. (2005). Phosphorylation and Regulation of Akt/PKB by the Rictor-mTOR Complex. Science.

[22] Philp A., Hamilton D.L., Baar K. (2011). Highlighted Topic Signals Mediating Skeletal Muscle Remodeling by Activity Signals Mediating Skeletal Muscle Remodeling by Resistance Exercise: PI3-Kinase Independent Activation of mTORC1. J. Appl. Physiol..

[23] Frey J.W., Jacobs B.L., Goodman C.A., Hornberger T.A. (2014). A Role for Raptor Phosphorylation in the Mechanical Activation of mTOR Signaling. Cell. Signal..

[24] You J.S., Mcnally R.M., Jacobs B.L., Privett R.E., Gundermann D.M., Lin K.H., Steinert N.D., Goodman C.A., Hornberger T.A. (2019). The Role of Raptor in the Mechanical Load-Induced Regulation of mTOR Signaling, Protein Synthesis, and Skeletal Muscle Hypertrophy. FASEB J..

[25] Vary T.C., Jefferson L.S., Kimball S.R. (1999). Amino Acid-Induced Stimulation of Translation Initiation in Rat Skeletal Muscle. Am. J. Physiol..

[26] Jeyapalan A.S., Orellana R.A., Suryawan A., O’Connor P.M.J., Nguyen H.V., Escobar J., Frank J.W., Davis T.A. (2007). Glucose Stimulates Protein Synthesis in Skeletal Muscle of Neonatal Pigs through an AMPK- and mTOR-Independent Process. Am. J. Physiol. Endocrinol. Metab..

[27] Sancak Y., Peterson T.R., Shaul Y.D., Lindquist R.A., Thoreen C.C., Bar-Peled L., Sabatini D.M. (2008). The Rag GTPases Bind Raptor and Mediate Amino Acid Signaling to mTORC1. Science.

[28] Korzick D.H., Sharda D.R., Pruznak A.M., Lang C.H. (2013). Aging Accentuates Alcohol-Induced Decrease in Protein Synthesis in Gastrocnemius. Am. J. Physiol. Regul. Integr. Comp. Physiol..

[29] Tang H., Inoki K., Brooks S.V., Okazawa H., Lee M., Wang J., Kim M., Kennedy C.L., Macpherson P.C.D., Ji X. (2019). mTORC1 Underlies Age-Related Muscle Fiber Damage and Loss by Inducing Oxidative Stress and Catabolism. Aging Cell.

[30] Cohen S., Zhai B., Gygi S.P., Goldberg A.L. (2012). Ubiquitylation by Trim32 Causes Coupled Loss of Desmin, Z-Bands, and Thin Filaments in Muscle Atrophy. J. Cell Biol..

[31] Mokhonova E.I., Avliyakulov N.K., Kramerova I., Kudryashova E., Haykinson M.J., Spencer M.J. (2015). The E3 Ubiquitin Ligase TRIM32 Regulates Myoblast Proliferation by Controlling Turnover of NDRG2. Hum. Mol. Genet..

[32] Skoglund E., Grönholdt-Klein M., Rullman E., Thornell L.E., Strömberg A., Hedman A., Cederholm T., Ulfhake B., Gustafsson T. (2020). Longitudinal Muscle and Myocellular Changes in Community-Dwelling Men over Two Decades of Successful Aging–The ULSAM Cohort Revisited. J. Gerontol. A Biol. Sci. Med. Sci..

[33] Kenyon C.J. (2010). The Genetics of Ageing. Nature.

[34] Sirago G., Picca A., Giacomello E., Marzetti E., Toniolo L. (2022). The Contribution of Genetics to Muscle Disuse, Retraining, and Aging. Genes.

[35] Segalés J., Perdiguero E., Serrano A.L., Sousa-Victor P., Ortet L., Jardí M., Budanov A.V., Garcia-Prat L., Sandri M., Thomson D.M. (2020). Sestrin Prevents Atrophy of Disused and Aging Muscles by Integrating Anabolic and Catabolic Signals. Nat. Commun..

[36] Gu X., Jouandin P., Lalgudi P.V., Binari R., Valenstein M.L., Reid M.A., Allen A.E., Kamitaki N., Locasale J.W., Perrimon N. (2022). Sestrin Mediates Detection of and Adaptation to Low-Leucine Diets in Drosophila. Nature.

[37] Xu D., Shimkus K.L., Lacko H.A., Kutzler L., Jefferson L.S., Kimball S.R. (2019). Evidence for a Role for Sestrin1 in Mediating Leucine-Induced Activation of mTORC1 in Skeletal Muscle. Am. J. Physiol. Endocrinol. Metab..

[38] Bentzinger C.F., Romanino K., Cloëtta D., Lin S., Mascarenhas J.B., Oliveri F., Xia J., Casanova E., Costa C.F., Brink M. (2008). Skeletal Muscle-Specific Ablation of Raptor, but Not of Rictor, Causes Metabolic Changes and Results in Muscle Dystrophy. Cell Metab..

[39] Shaw R.J. (2008). Raptor Swoops in on Metabolism. Cell Metab..

[40] Shu L., Houghton P.J. (2009). The MTORC2 Complex Regulates Terminal Differentiation of C2C12 Myoblasts. Mol. Cell. Biol..

[41] Ruiz A., Dror E., Handschin C., Furrer R., Perez-Schindler J., Bachmann C., Treves S., Zorzato F. (2018). Over-Expression of a Retinol Dehydrogenase (SRP35/DHRS7C) in Skeletal Muscle Activates mTORC2, Enhances Glucose Metabolism and Muscle Performance. Sci. Rep..

[42] Kowalsky A.H., Namkoong S., Mettetal E., Park H.W., Kazyken D., Fingar D.C., Lee J.H. (2020). The GATOR2-mTORC2 Axis Mediates Sestrin2-Induced AKT Ser/Thr Kinase Activation. J. Biol. Chem..

[43] Rion N., Castets P., Lin S., Enderle L., Reinhard J.R., Ruëgg M.A. (2019). mTORC2 Affects the Maintenance of the Muscle Stem Cell Pool. Skelet. Muscle.

[44] Sanvee G.M., Hitzfeld L., Bouitbir J., Krähenbühl S. (2021). mTORC2 Is an Important Target for Simvastatin-Associated Toxicity in C2C12 Cells and Mouse Skeletal Muscle–Roles of Rap1 Geranylgeranylation and Mitochondrial Dysfunction. Biochem. Pharmacol..

[45] Fry C.S., Drummond M.J., Glynn E.L., Dickinson J.M., Gundermann D.M., Timmerman K.L., Walker D.K., Dhanani S., Volpi E., Rasmussen B.B. (2011). Aging Impairs Contraction-Induced Human Skeletal Muscle mTORC1 Signaling and Protein Synthesis. Skelet. Muscle.

[46] Sandri M., Barberi L., Bijlsma A.Y., Blaauw B., Dyar K.A., Milan G., Mammucari C., Meskers C.G.M., Pallafacchina G., Paoli A. (2013). Signalling Pathways Regulating Muscle Mass in Ageing Skeletal Muscle. the Role of the IGF1-Akt-mTOR-FoxO Pathway. Biogerontology.

[47] Barns M., Gondro C., Tellam R.L., Radley-Crabb H.G., Grounds M.D., Shavlakadze T. (2014). Molecular Analyses Provide Insight into Mechanisms Underlying Sarcopenia and Myofibre Denervation in Old Skeletal Muscles of Mice. Int. J. Biochem. Cell Biol..

[48] Gaster M., Poulsen P., Handberg A., Schrøder H.D., Beck-Nielsen H. (2000). Direct Evidence of Fiber Type-Dependent GLUT-4 Expression in Human Skeletal Muscle. Am. J. Physiol. Endocrinol. Metab..

[49] Petersen K.F., Morino K., Alves T.C., Kibbey R.G., Dufour S., Sono S., Yoo P.S., Cline G.W., Shulman G.I. (2015). Effect of Aging on Muscle Mitochondrial Substrate Utilization in Humans. Proc. Natl. Acad. Sci. USA.

[50] Anand A., Nambirajan A., Kumar V., Agarwal S., Sharma S., Mohta S., Gopi S., Kaushal K., Gunjan D., Singh N. (2022). Alterations in Autophagy and Mammalian Target of Rapamycin (mTOR) Pathways Mediate Sarcopenia in Patients with Cirrhosis. J. Clin. Exp. Hepatol..

[51] Barnard R.J., Youngren J.F., Martin D.A. (1995). Diet, Not Aging, Causes Skeletal Muscle Insulin Resistance. Gerontology.

[52] Bian A., Ma Y., Zhou X., Guo Y., Wang W., Zhang Y., Wang X. (2020). Association between Sarcopenia and Levels of Growth Hormone and Insulin-like Growth Factor-1 in the Elderly. BMC Musculoskelet. Disord..

[53] Chew J., Tay L., Lim J.P., Leung B.P., Yeo A., Yew S., Ding Y.Y., Lim W.S. (2019). Serum Myostatin and IGF-1 as Gender-Specific Biomarkers of Frailty and Low Muscle Mass in Community-Dwelling Older Adults. J. Nutr. Health Aging.

[54] Roubenoff R., Parise H., Payette H.A., Abad L.W., D’Agostino R., Jacques P.F., Wilson P.W.F., Dinarello C.A., Harris T.B. (2003). Cytokines, Insulin-like Growth Factor 1, Sarcopenia, and Mortality in Very Old Community-Dwelling Men and Women: The Framingham Heart Study. Am. J. Med..

[55] Kwak J.Y., Hwang H., Kim S.K., Choi J.Y., Lee S.M., Bang H., Kwon E.S., Lee K.P., Chung S.G., Kwon K.S. (2018). Prediction of Sarcopenia Using a Combination of Multiple Serum Biomarkers. Sci. Rep..

[56] Deschenes M.R., McCoy R.W., Holdren A.N., Eason M.K. (2009). Gender Influences Neuromuscular Adaptations to Muscle Unloading. Eur. J. Appl. Physiol..

[57] Phillips B.E., Williams J.P., Gustafsson T., Bouchard C., Rankinen T., Knudsen S., Smith K., Timmons J.A., Atherton P.J. (2013). Molecular Networks of Human Muscle Adaptation to Exercise and Age. PLoS Genet..

[58] Farnfield M.M., Breen L., Carey K.A., Garnham A., Cameron-Smith D. (2012). Activation of MTOR Signalling in Young and Old Human Skeletal Muscle in Response to Combined Resistance Exercise and Whey Protein Ingestion. Appl. Physiol. Nutr. Metab..

[59] Drummond M.J., Miyazaki M., Dreyer H.C., Pennings B., Dhanani S., Volpi E., Esser K.A., Rasmussen B.B. (2009). Expression of Growth-Related Genes in Young and Older Human Skeletal Muscle Following an Acute Stimulation of Protein Synthesis. J. Appl. Physiol..

[60] Drummond M.J., Dreyer H.C., Pennings B., Fry C.S., Dhanani S., Dillon E.L., Sheffield-Moore M., Volpi E., Rasmussen B.B. (2008). Skeletal Muscle Protein Anabolic Response to Resistance Exercise and Essential Amino Acids Is Delayed with Aging. J. Appl. Physiol..

[61] Figueiredo V.C., Englund D.A., Vechetti I.J., Alimov A., Peterson C.A., McCarthy J.J. (2019). Phosphorylation of Eukaryotic Initiation Factor 4E Is Dispensable for Skeletal Muscle Hypertrophy. Am. J. Physiol. Cell Physiol..

[62] Rieger F., Grumet M., Edelman G.M. (1985). N-CAM at the Vertebrate Neuromuscular Junction. J. Cell Biol..

[63] Dickson G., Gower H.J., Barton C.H., Prentice H.M., Elsom V.L., Moore S.E., Cox R.D., Quinn C., Putt W., Walsh F.S. (1987). Human Muscle Neural Cell Adhesion Molecule (N-CAM): Identification of a Muscle-Specific Sequence in the Extracellular Domain. Cell.

[64] Markofski M.M., Dickinson J.M., Drummond M.J., Fry C.S., Fujita S., Gundermann D.M., Glynn E.L., Jennings K., Paddon-Jones D., Reidy P.T. (2015). Effect of Age on Basal Muscle Protein Synthesis and mTORC1 Signaling in a Large Cohort of Young and Older Men and Women. Exp. Gerontol..

[65] Paturi S., Gutta A.K., Katta A., Kakarla S.K., Arvapalli R.K., Gadde M.K., Nalabotu S.K., Rice K.M., Wu M., Blough E. (2010). Effects of Aging and Gender on Muscle Mass and Regulation of Akt-mTOR-P70s6k Related Signaling in the F344BN Rat Model. Mech. Ageing Dev..

[66] White Z., White R.B., McMahon C., Grounds M.D., Shavlakadze T. (2016). High mTORC1 Signaling Is Maintained, While Protein Degradation Pathways Are Perturbed in Old Murine Skeletal Muscles in the Fasted State. Int. J. Biochem. Cell Biol..

[67] Cuthbertson D.J., Babraj J., Leese G., Siervo M. (2017). Anabolic Resistance Does Not Explain Sarcopenia in Patients with Type 2 Diabetes Mellitus, Compared with Healthy Controls, despite Reduced mTOR Pathway Activity. Clin. Nutr..

[68] Kimball S.R., O’Malley J.P., Anthony J.C., Crozier S.J., Jefferson L.S. (2004). Assessment of Biomarkers of Protein Anabolism in Skeletal Muscle during the Life Span of the Rat: Sarcopenia despite Elevated Protein Synthesis. Am. J. Physiol. Endocrinol. Metab..

[69] Marabita M., Baraldo M., Solagna F., Ceelen J.J.M., Sartori R., Nolte H., Nemazanyy I., Pyronnet S., Kruger M., Pende M. (2016). S6K1 Is Required for Increasing Skeletal Muscle Force during Hypertrophy. Cell Rep..

[70] Le Bacquer O., Combe K., Patrac V., Ingram B., Combaret L., Dardevet D., Montaurier C., Salles J., Giraudet C., Guillet C. (2019). 4E-BP1 and 4E-BP2 Double Knockout Mice Are Protected from Aging-Associated Sarcopenia. J. Cachexia Sarcopenia Muscle.

[71] Chen Z., Li L., Wu W., Liu Z., Huang Y., Yang L., Luo Q., Chen J., Hou Y., Song G. (2020). Exercise Protects Proliferative Muscle Satellite Cells against Exhaustion via the Igfbp7-Akt-mTOR Axis. Theranostics.

[72] De Sanctis P., Filardo G., Abruzzo P.M., Astolfi A., Bolotta A., Indio V., Di Martino A., Hofer C., Kern H., Löfler S. (2021). Non-Coding RNAs in the Transcriptional Network That Differentiates Skeletal Muscles of Sedentary from Long-Term Endurance-and Resistance-Trained Elderly. Int. J. Mol. Sci..

[73] Clavel S., Coldefy A.S., Kurkdjian E., Salles J., Margaritis I., Derijard B. (2006). Atrophy-Related Ubiquitin Ligases, Atrogin-1 and MuRF1 Are up-Regulated in Aged Rat Tibialis Anterior Muscle. Mech. Ageing Dev..

[74] Edström E., Altun M., Hägglund M., Ulfhake B. (2006). Atrogin-1/MAFbx and MuRF1 Are Downregulated in Aging-Related Loss of Skeletal Muscle. J. Gerontol. A Biol. Sci. Med. Sci..

[75] DeRuisseau K.C., Kavazis A.N., Powers S.K. (2005). Selective Downregulation of Ubiquitin Conjugation Cascade mRNA Occurs in the Senescent Rat Soleus Muscle. Exp. Gerontol..

[76] Welle S., Brooks A.I., Delehanty J.M., Needler N., Thornton C.A. (2003). Gene Expression Profile of Aging in Human Muscle. Physiol. Genom..

[77] Whitman S.A., Wacker M.J., Richmond S.R., Godard M.P. (2005). Contributions of the Ubiquitin-Proteasome Pathway and Apoptosis to Human Skeletal Muscle Wasting with Age. Pflug. Arch. Eur. J. Physiol..

[78] Léger B., Derave W., De Bock K., Hespel P., Russell A.P. (2008). Human Sarcopenia Reveals an Increase in SOCS-3 and Myostatin and a Reduced Efficiency of Akt Phosphorylation. Rejuvenation Res..

[79] Gaugler M., Brown A., Merrell E., DiSanto-Rose M., Rathmacher J.A., Reynolds IV T.H. (2011). PKB Signaling and Atrogene Expression in Skeletal Muscle of Aged Mice. J. Appl. Physiol..

[80] Woo Kim K., Cho H.J., Khaliq S.A., Son K.H., Yoon M.S. (2020). Comparative Analyses of mTOR/Akt and Muscle Atrophy-Related Signaling in Aged Respiratory and Gastrocnemius Muscles. Int. J. Mol. Sci..

[81] Liu X., Zhang Y., Sun X., Zhang W., Shi X., Xu S. (2022). Di-(2-Ethyl Hexyl) Phthalate Induced Oxidative Stress Promotes Microplastics Mediated Apoptosis and Necroptosis in Mice Skeletal Muscle by Inhibiting PI3K/AKT/mTOR Pathway. Toxicology.

[82] Zeng Z., Liang J., Wu L., Zhang H., Lv J., Chen N. (2020). Exercise-Induced Autophagy Suppresses Sarcopenia Through Akt/mTOR and Akt/FoxO3a Signal Pathways and AMPK-Mediated Mitochondrial Quality Control. Front. Physiol..

[83] Morisasa M., Yoshida E., Fujitani M., Kimura K., Uchida K., Kishida T., Mori T., Goto-Inoue N. (2022). Fish Protein Promotes Skeletal Muscle Hypertrophy via the Akt/mTOR Signaling Pathways. J. Nutr. Sci. Vitaminol..

[84] Liu Y., Wang Q., Zhang Z., Fu R., Zhou T., Long C., He T., Yang D., Li Z., Peng S. (2021). Magnesium Supplementation Enhances mTOR Signalling to Facilitate Myogenic Differentiation and Improve Aged Muscle Performance. Bone.

[85] Aoi W., Iwasa M., Aiso C., Tabata Y., Gotoh Y., Kosaka H., Suzuki T. (2022). Lactococcus Cremoris Subsp. Cremoris FC-Fermented Milk Activates Protein Synthesis and Increases Skeletal Muscle Mass in Middle-Aged Mice. Biochem. Biophys. Res. Commun..

[86] Chen C.N., Liao Y.H., Tsai S.C., Thompson L.D.V. (2019). Age-Dependent Effects of Caloric Restriction on mTOR and Ubiquitin-Proteasome Pathways in Skeletal Muscles. GeroScience.

[87] Toniolo L., Formoso L., Torelli L., Crea E., Bergamo A., Sava G., Giacomello E. (2021). Long-Term Resveratrol Treatment Improves the Capillarization in the Skeletal Muscles of Ageing C57BL/6J Mice. Int. J. Food Sci. Nutr..

[88] Sirago G., Toniolo L., Crea E., Giacomello E. (2022). A Short-Term Treatment with Resveratrol Improves the Inflammatory Conditions of Middle-Aged Mice Skeletal Muscles. Int. J. Food Sci. Nutr..

[89] Toniolo L., Fusco P., Formoso L., Mazzi A., Canato M., Reggiani C., Giacomello E. (2018). Resveratrol Treatment Reduces the Appearance of Tubular Aggregates and Improves the Resistance to Fatigue in Aging Mice Skeletal Muscles. Exp. Gerontol..

[90] Barger J.L., Kayo T., Vann J.M., Arias E.B., Wang J., Hacker T.A., Wang Y., Raederstorff D., Morrow J.D., Leeuwenburgh C. (2008). A Low Dose of Dietary Resveratrol Partially Mimics Caloric Restriction and Retards Aging Parameters in Mice. PLoS ONE.

[91] Dutta D., Xu J., Dirain M.L.S., Leeuwenburgh C. (2014). Calorie Restriction Combined with Resveratrol Induces Autophagy and Protects 26-Month-Old Rat Hearts from Doxorubicin-Induced Toxicity. Free Radic. Biol. Med..

[92] Opalach K., Rangaraju S., Madorsky I., Leeuwenburgh C., Notterpek L. (2010). Lifelong Calorie Restriction Alleviates Age-Related Oxidative Damage in Peripheral Nerves. Rejuvenation Res..

[93] Picca A., Fracasso F., Pesce V., Cantatore P., Joseph A.M., Leeuwenburgh C., Gadaleta M.N., Lezza A.M.S. (2013). Age-and Calorie Restriction-Related Changes in Rat Brain Mitochondrial DNA and TFAM Binding. Age.

[94] Chimienti G., Picca A., Fracasso F., Russo F., Orlando A., Riezzo G., Leeuwenburgh C., Pesce V., Lezza A.M.S. (2021). The Age-Sensitive Efficacy of Calorie Restriction on Mitochondrial Biogenesis and Mtdna Damage in Rat Liver. Int. J. Mol. Sci..

[95] Gharahdaghi N., Rudrappa S., Brook M.S., Idris I., Crossland H., Hamrock C., Aziz M.H.A., Kadi F., Tarum J., Greenhaff P.L. (2019). Testosterone Therapy Induces Molecular Programming Augmenting Physiological Adaptations to Resistance Exercise in Older Men. J. Cachexia Sarcopenia Muscle.

[96] Kim J.E., Kwon E.Y., Han Y. (2022). A Collagen Hydrolysate Containing Tripeptides Ameliorates Sarcopenia in Middle-Aged Mice. Molecules.

[97] Baek M.O., Cho H.J., Min D.S., Choi C.S., Yoon M.S. (2022). Self-Transducible LRS-UNE-L Peptide Enhances Muscle Regeneration. J. Cachexia Sarcopenia Muscle.

[98] Yi D., Yoshikawa M., Sugimoto T., Tomoo K., Okada Y., Hashimoto T. (2022). Effects of Maca on Muscle Hypertrophy in C2C12 Skeletal Muscle Cells. Int. J. Mol. Sci..

[99] Le Bacquer O., Lanchais K., Combe K., Van Den Berghe L., Walrand S. (2021). Acute Rimonabant Treatment Promotes Protein Synthesis in C2C12 Myotubes through a CB1-Independent Mechanism. J. Cell. Physiol..

[100] Kwon D., Kim C., Woo Y.K., Hwang J.K. (2021). Inhibitory Effects of Chrysanthemum (*Chrysanthemum Morifolium* Ramat.) Extract and Its Active Compound Isochlorogenic Acid A on Sarcopenia. Prev. Nutr. Food Sci..

